# Supplementary material for: Longitudinal analysis of the rectal microbiome in dogs with diabetes mellitus after initiation of insulin therapy
Source: PLoS One. 2022 Sep 6;17(9):e0273792. doi: 10.1371/journal.pone.0273792 (PMC9447884; doi:10.1371/journal.pone.0273792)
Supplement: S1 File — Separate questionnaires were developed for the first (time 0) and subsequent (time 2–12) visits to assess owner and veterinarian perceptions of control of clinical signs of diabetes mellitus. (DOCX) [file pone.0273792.s001.docx]

**OWNER Canine DIABETIC Fecal Microbiome Study Form** – Week 0 Study Number ________

Patient Name ______________________________________________________

Patient Clinic Number _________________________ Today’s Date ______________________

Age ________ Sex _______ Neutered/Spayed?_____ Breed ____________________

When was your dog diagnosed with diabetes? ______________________________

What kind of food does your dog eat? ______________________________________

Compared to before your dog became diabetic, how is your dog’s water intake? (circle one)

DECREASED NORMAL MILDLY INCREASED VERY INCREASED

Compared to before your dog became diabetic, how is your dog’s urination frequency/volume?

DECREASED NORMAL MILDLY INCREASED VERY INCREASED

Compared to before your dog became diabetic, how is your dog’s appetite? (circle one)

DECREASED NORMAL MILDLY INCREASED VERY INCREASED

Compared to before your dog became diabetic, how is your dog’s weight/body condition? (circle one)

VERY DECREASED DECREASED NORMAL INCREASED

Compared to before your dog became diabetic, how is your dog’s activity level? (circle one)

VERY DECREASED DECREASED NORMAL INCREASED

Does your dog have other medical conditions? If so, please list: __________________________________________________________________________________________________________________________________________________________________________________

Other than insulin, is your dog on other medications? If so, please list: __________________________________________________________________________________________________________________________________________________________________________________

Has your dog been on any antibiotic medications in the past 30 days? YES NO

If needed, may we contact you or your vet for additional information? YES NO

**OWNER Canine DIABETIC Fecal Microbiome Study Form** – Weeks 2-12 Study Number ________

Patient Name ______________________________________________________

Patient Clinic Number _________________________ Today’s Date ______________________

Age ________ Sex _______ Neutered/Spayed?_____ Breed ____________________

When was your dog diagnosed with diabetes? ______________________________

What kind of insulin does your dog currently use? ___________________________________

How many units? ________________ ONCE or TWICE daily? (circle one)

How long has your dog been on this type/dose of insulin? ____________________________

What kind of food does your dog eat? ______________________________________

Compared to before your dog became diabetic, how is your dog’s water intake? (circle one)

DECREASED NORMAL MILDLY INCREASED VERY INCREASED

Compared to before your dog became diabetic, how is your dog’s urination frequency/volume?

DECREASED NORMAL MILDLY INCREASED VERY INCREASED

Compared to before your dog became diabetic, how is your dog’s appetite? (circle one)

DECREASED NORMAL MILDLY INCREASED VERY INCREASED

Compared to before your dog became diabetic, how is your dog’s weight/body condition? (circle one)

VERY DECREASED DECREASED NORMAL INCREASED

Compared to before your dog became diabetic, how is your dog’s activity level? (circle one)

VERY DECREASED DECREASED NORMAL INCREASED

Does your dog have other medical conditions? If so, please list: __________________________________________________________________________________________________________________________________________________________________________________

Other than insulin, is your dog on other medications? If so, please list: __________________________________________________________________________________________________________________________________________________________________________________

**VETERINARIAN Canine DIABETIC Fecal Microbiome Study Form** – Week 0 Study Number ________

Patient Name ______________________________________________________

Patient Clinic Number _________________________ Today’s Date ______________________

Age ________ Sex _______ Neutered/Spayed?_____ Breed _____________________________

Veterinarian’s Name __________________________________________________

What is the dog’s weight today? ______________ kg/lbs

What/when was the dog’s last recorded weight? ______________ kg/lbs Date _______________

On a nine-point scale, what is this dog’s body condition score? ______________________

Please estimate the dog’s ideal body weight ______________ kg/lbs

Describe any muscle wasting: NONE MILD MODERATE SEVERE (circle one)

Please list any pertinent physical exam findings:

__________________________________________________________________________________________________________________________________________________________________________________

_________________________________________________________________________________________

Please briefly summarize the dog’s presenting complaint and history (if known):

__________________________________________________________________________________________________________________________________________________________________________________

_________________________________________________________________________________________

_________________________________________________________________________________________

Please verify that this dog does not have any of the following:

| Yes | No | Diabetic ketoacidosis or hyperglycemic hyperosmolar syndrome |
| --- | --- | --- |
| Yes | No | Hyperadrenocorticism, hypoadrenocorticism, hypothyroidism |
| Yes | No | Inflammatory bowel disease/idiopathic chronic enteropathy |
| Yes | No | Exocrine pancreatic insufficiency |
| Yes | No | Chronic hepatitis |
| Yes | No | Immune-mediated anemia/thrombocytopenia/polyarthropathy |
| Yes | No | Infectious disease |
| Yes | No | Neoplasia |
| Yes | No | Has not had diet change in past 30 days |
| Yes | No | Has not received antibiotics in past 30 days |

Please list what insulin type, dose, and frequency you are prescribing: ______________________________

**VETERINARIAN Canine DIABETIC Fecal Microbiome Study Form** Weeks #2-12

Study Number ________

Patient Name ______________________________________________________

Patient Clinic Number _________________________ Today’s Date ______________________

Age ________ Sex _______ Neutered/Spayed?_____ Breed _____________________________

Veterinarian’s Name __________________________________________________

What is the dog’s weight today? ______________ kg/lbs

On a nine-point scale, what is this dog’s body condition score? ______________________

Please estimate the dog’s ideal body weight ______________ kg/lbs

Describe any muscle wasting: NONE MILD MODERATE SEVERE (circle one)

Please list any pertinent physical exam findings:

________________________________________________________________________________________________________________________________________________________________________________________________________________________________________________________________________________________________________________________________________________________________________

Did this visit result in recommendation for hospitalization or treatment for any diabetes-related complications (e.g. diabetic ketoacidosis, hyperglycemic-hyperosmolar syndrome, urinary tract infection)? If yes, please explain:

________________________________________________________________________________________________________________________________________________________________________________________________________________________________________________________________________________________________________________________________________________________________________

Please briefly summarize the dog’s diabetes treatment history since last visit and changes made today: ________________________________________________________________________________________________________________________________________________________________________________________________________________________________________________________________________________________________________________________________________________________________________

Please verify the dog’s current insulin type, dose, and frequency: ____________________________________

In your clinical opinion, how would you rate this dog’s diabetic regulation?

POORLY CONTROLLED MODERATELY CONTROL WELL CONTROLLED
