# Supplementary material for: Longitudinal analysis of the rectal microbiome in dogs with diabetes mellitus after initiation of insulin therapy
Source: PLoS One. 2022 Sep 6;17(9):e0273792. doi: 10.1371/journal.pone.0273792 (PMC9447884; doi:10.1371/journal.pone.0273792)
Supplement: Attachment — Submitted filename: Review.docx [file pone.0273792.s002.docx]

Longitudinal analysis of the rectal microbiome in dogs with diabetes mellitus after initiation of insulin therapy

Line 91 *Enterobacteriaceae* should be italicized

Line 216 As should be added before per

Line 264 “was a large and significant” should be reframed

Line 267 “from” to be removed

Line 282 Please add full stop

Line 320 Instead of Diabetic it should be Insulin

Line 353 Check spelling of *Enterobacteriaceae*

Line 359 Please see “in”

Line 381 Replace Methods with method

Line 398 Studies.

There are few questions:

1. The authors said

“The objective of this pilot study was to characterize the gastrointestinal microbiome of dogs with diabetes mellitus at the time of diagnosis and over the first 12 weeks of insulin therapy and identify associations with glycemic control”

However they are also suggesting that

“This pilot study was not designed to make inferences about cause and effect between the intestinal microbial composition and host health; however, it does demonstrate shifts within the intestinal microbiome of dogs with diabetes during initial insulin therapy”.

So, if, the objective was not to make any inference of cause and effect between the intestinal microbial composition and host health then what is the purpose of this study?

2. In this study statistically significant changes in microbial diversity were not observed thus it means that the microbiome did not change significantly before and after the initiation of the insulin therapy. Does it mean that there is no significant role of microbial diversity in DM in dogs.
